# P093: Impact of antimicrobial restriction program on antimicrobial agents usage

**DOI:** 10.1186/2047-2994-2-S1-P93

**Published:** 2013-06-20

**Authors:** HK Ki, H-S Cheong

**Affiliations:** 1 Internal Medicine, Seoul, Korea, Republic Of; 2 KonKuk University Hospital, Seoul, Korea, Republic Of

## Introduction

Antimicrobial agents have been used inappropriately so far. Recently antimicrobial stewardship has been stressed for the prevention of spread of antimicrobial resistant organism. Especially antimicrobial restriction before usage has been used in many institutions. The impact of the antimicrobial restriction has not been known adequately.

## Objectives

The goal of the study is to know the impact of antimicrobial restriction program on the pattern of antimicrobial prescription and antimicrobial resistance.

## Methods

We reviewed the prescribed antimicrobial agents and the dosage of each antimicrobial agents from the year 2005 to year 2012. We collect the data using computerized antimicrobial usage program.

## Results

Antimicrobial restriction program has been launched from the year 2006. ID physicians previewed the necessity of the antimicrobial agents before the usage. The restricted formula are as followed : 3rd / 4th genenration cephalosporin, carbapenem, fluoroquinolone, glycopeptide, aminoglycoside, and antifungals. The average annual prescription amount was 770,563 DDD (Range 650,225-1,109,740 DDD). The total amount of prescribed antimicrobial agents showed plauteau from year 2009. Third generation cephalosporin (34,531 DDD to 22,772 DDD) and aminoglycoside (4,557 DDD to 2,688 DDD) were less prescribed after introduction of formula restriction. Quinolone class are also less prescribed (25,521 DDD to 9,662 DDD). While, penicillin (4,500 DDD to 34,034 DDD) and first generation cephalosporin (4,166 DDD to 15,194 DDD) were more prescribed after introduction of formula restriction.

## Conclusion

Total amount of antimicrobial presciption showed plauteau after introduction of antimicrobial restriction program. Third generation cephalosporin, Aminoglycoside, and quinolone were less prescibed than before. Antimicrobial restriction program wound not increase the total amount of prescription and even decrease the usage of broad spectrum antimicrobial agents.

## Disclosure of interest

None declared.

